# Regulation of p73 by Pirh2-AIP4 loop

**DOI:** 10.18632/aging.203537

**Published:** 2021-09-14

**Authors:** H. Helena Wu, Rami Abou Zeinab, Roger P. Leng

**Affiliations:** 1 Heritage Medical Research Center, Department of Laboratory Medicine and Pathology, University of Alberta, Edmonton, Alberta T6G 2S2, Canada

**Keywords:** Pirh2, AIP4, p73, ubiquitination

Ubiquitination and proteasome degradation play a vital role in controlling the level and activity of a protein. The protein p73 has been identified as a homolog of the tumor suppressor protein p53 and is capable of inducing apoptosis or cell cycle arrest [1]. Nonetheless, p73 is rarely found to be mutated in human tumors, and p73-deficient mice display no increase in tumor incidence. However, several studies have shown that p73 is missing or reduced in expression in certain human tumors (through loss of heterozygosity or allele silencing), thus implying that p73 has tumor-suppressive activities. Flores et al. have shown that p73^+/-^ mice develop spontaneous tumors, that the loss of p73 can cooperate with p53 in tumor suppression, and that simultaneous mutation of both p73 and p53 leads to a more aggressive tumor phenotype, thus indicating that p73 plays an important role in tumor suppression in the mouse model [2]. This suggests that the regulation of p73 expression and stability are essential for maintaining normal cell growth. Several ubiquitin E3 ligases have been reported to regulate p73 stability; however, the mechanism of p73 regulation remains partially understood. Atrophin-1 interacting protein 4 (AIP4, also called Itch) contains an N-terminal C2 domain, protein interacting WW domains, and a C-terminal HECT ligase domain, which belongs to HECT (homologous to the E6-AP Carboxyl Terminus) domain proteins. It was reported that AIP4 serves as an E3 ligase for Notch, CXCR4, LMP2A, HEF1, Smad2, ErbB-4, p73, p63 [3]. AIP4 promotes the ubiquitination and degradation of p73 [4]. AIP4 inhibits p73 dependent transcriptional activity. Interestingly, AIP4 shows no E3 ligase activity towards p53 despite the homology between the two tumor suppressors [4]. Pirh2 is a RING finger ligase that binds to and promotes p53 degradation [5]. Furthermore, Pirh2 downregulates p53-dependent transactivation and p53-mediated inhibition of cell growth, and like Mdm2, Pirh2 participates in an auto-regulatory feedback loop involving p53 [5]. Overexpression of Pirh2 in mouse tumors is accompanied by low p53 protein expression, but Mdm2 levels remain unchanged. E3 ligases often control the ubiquitination of multiple substrates; Pirh2 serves as an E3 ligase for AR, Tip60, hNTKL-BP1, p27^Kip1^, p73, p63, CHK2 [6]. It was reported that Pirh2 deficiency mice resulted in higher levels of p53 in several tissues in response to DNA damage and despite the presence of other E3 ligases for p53 [7]. Pirh2 is vital for the in vivo regulation of p53 stability in response to DNA damage. Hakem et al. found that Pirh2 interacted with c-Myc, and mediated its polyubiquitination and proteolysis [7]. The two apparently opposite functions of Pirh2, which promotes or inhibits tumorigenesis, are intriguing and suggest a novel cell-growth-regulatory mechanism. It is possible that both the functions are necessary at different stages of normal cell growth, and a balance of the two functions is maintained in normal cells. We recently found that Pirh2 and AIP4 physically interacted in cells and in vitro [8]. Interestingly, overexpression of Pirh2 decreased the AIP4 level, whereas AIP4 upregulated Pirh2 expression. This result is considered the basis for speculating on the existence of a regulatory mechanism between Pirh2 and AIP4 in terms of p73 regulation. Pirh2 promotes the ubiquitination and degradation of AIP4. We mapped the Pirh2 and AIP4 interaction domains. HECT domain in AIP4 is involved in Pirh2 binding. The deletion of the HECT domain inhibited AIP4-Pirh2 binding, thus confirming its role in the binding mechanism. However, the HECT domain has been shown to have no effect on AIP4-p73 binding [4]. We confirmed the binding of both ligases, Pirh2 and AIP4, in cells and in vitro, and that the HECT domain of AIP4 is required for Pirh2 interaction; however, the N-terminal domain (NTD) of Pirh2 is required for AIP4 interaction [8]. Notably, the key to the catalytic activity of HECT E3 ligases is the conformational flexibility conferred by a hinge-like region within the bilobed HECT structure. This flexible hinge region is critical for juxtaposing the catalytic cysteine residues of the E2 and HECT domain during ubiquitin transfer. It is possible that Pirh2 and HECT of AIP4 directly interacted and that preventing such flexibility within the HECT domain will greatly reduce its activity for p73 (or other substrates). The regulatory mechanism was further defined as Pirh2 decreased AIP4–p73 negative regulation. In summary, we have discovered two proteins, Pirh2 and AIP4, that form a feedback loop to regulate p73. Cancers are considered an age-related disease. p73 has been linked to age-related diseases. Our study reveals a novel link between two E3 ligases previously thought to be unrelated in regulating the same effector substrate, p73. By identifying genes and biological pathways that contribute to human cancers, our research has the potential to identify new therapeutic targets.
